# The role of grit in inclusive education: a study of motivation and achievement among preservice physical education teachers

**DOI:** 10.3389/fpsyg.2024.1332464

**Published:** 2024-01-29

**Authors:** Joonyoung Lee, Jinwoo Park

**Affiliations:** ^1^Department of Health, Physical Education, and Recreation, Jackson State University, Jackson, MS, United States; ^2^Sports Science Institute, Pusan National University, Busan, Republic of Korea

**Keywords:** grit, preservice PE teachers, motivation, achievement goals, career intentions, structural equation modeling, inclusive education, physical education

## Abstract

**Introduction:**

Grit, a combination of enduring effort and persistent interest, is key to long-term goals. The training of preservice physical education (PE) teachers is vital for child development, emphasizing the need to assess their resilience and commitment. However, research is limited regarding how grit influences motivation and achievement goals in PE. The purpose of this study was to explore how the grit dimensions of preservice PE teachers impact their motivation and achievement goals, which may subsequently shape their future career intentions of becoming PE teachers.

**Methods:**

A total of 279 preservice physical education (PE) teachers (69.5% males; 26.9% PE graduate program) from five South Korean universities participated in the study. They completed validated questionnaires measuring grit, motivation, achievement goal orientations, and career intentions. Descriptive statistics, correlation analysis, and structural equation modeling (SEM) were used to examine variable relationships and test the hypothesis model.

**Results:**

Correlation analysis indicated a spectrum of relationships between facets of grit (perseverance of effort and consistency of interests), motivational parameters, and career intention, with both positive and negative correlations ranging from weak to moderate (*r* ranging from 0.119 to 0.425, *p* < 0.05–0.01). SEM confirmed the model’s goodness-of-fit (χ^2^/df = 1.928, RMSEA = 0.058, IFI = 0.92, TLI = 0.91, CFI = 0.92). Path analysis showed that both perseverance of effort and consistency of interests significantly influenced motivational mechanisms (*β* ranging from −0.34 to 0.57, *p* < 0.05–0.01), both directly and indirectly, which then notably impacted career intentions (*β* = 0.10, *p* < 0.05). Notably, both grit dimensions significantly impacted mastery approach goals (*β* ranging from 0.49 to 0.56, *p* < 0.01). Mastery approach goals, in turn, had a substantial impact on intrinsic motivation (*β* = 0.27, *p* < 0.01), which subsequently significantly influenced career intentions (*β* = 0.32, *p* < 0.01).

**Conclusion:**

The study illuminated the complex relationships between grit dimensions, motivation, achievement goals, and career intentions of future PE teachers. SEM validation confirmed grit’s direct and indirect influence on goal orientations and motivation, underscoring the importance of incorporating grit-building strategies alongside mastery approach goals in preservice PE programs to enhance resilience, dedication, and long-term career commitment.

## 1 Introduction

Physical education (PE) is a crucial component of K-12 education, promoting physical fitness, motor skills development, overall health, and well-being while also enhancing social–emotional learning and cognitive abilities for a balanced education (Centers for Disease Control and Prevention, 2022; Society of Health and Physical Educators [SHAPE America], 2022). Given PE’s significance within the K-12 curriculum, PE teacher education (PETE) for preservice PE teachers (PPETs) is increasingly vital as an essential aspect of teacher preparation, ensuring they are well-equipped to effectively and successfully implement PE programs in schools, ultimately fostering whole child development (Silverman and Mercier, 2015; McEvoy et al., 2017; Webster et al., 2017; Lee et al., 2021). The importance of PETE programs in fostering the education of PPETs cannot be overstated. Hence, it is crucial to monitor their perseverance and passion toward achieving long-term goals, such as becoming PE teachers in the near future. This tenacity is closely associated with the concept of “grit.”

Individuals with grit have the ability to maintain their concentration and determination over prolonged periods, despite facing obstacles or setbacks (Duckworth et al., 2011). The concept of grit gained prominence through the work of psychologist Dr. Angela Duckworth, who has emphasized its importance in achieving success beyond just talent or intelligence (Duckworth et al., 2007, 2011; Duckworth and Quinn, 2009). Grit is conceptualized as comprising two distinct dimensions: *perseverance of effort* (e.g., resilience when confronting obstacles) and *consistency of interests* (e.g., enduring commitments over an extended period; Duckworth et al., 2007). Previous studies have revealed two distinct facets of grit showing that while perseverance of effort was more strongly related to academic outcomes (i.e., performance, engagement) and well-being (Datu et al., 2016; Credé et al., 2017; Muenks et al., 2018; Disabato et al., 2019; Karlen et al., 2019; Tang et al., 2019), consistency of interests was more closely associate with engagements in physical behaviors and psychological coping skills (Hein et al., 2019; Newland et al., 2020). Recognized as a critical element in both personal and professional spheres, the grit dimensions facilitate individuals’ capacity to navigate challenges and surmount difficulties. Longitudinal evidence also demonstrated that grit dimensions progressively contributed to predicting success in a diverse range of academic achievements, such as retention, educational achievements, and teaching effectiveness (Duckworth et al., 2009; Robertson-Kraft and Duckworth, 2014).

Grit has been shown to enhance individuals’ motivation and their pursuit of achievement goals. Duckworth and colleagues’ research has been pivotal in highlighting that individuals with higher levels of grit dimensions tend to maintain motivation and resilience even in adverse circumstances (Duckworth et al., 2007, 2009). The finding is further supported by studies that identify effort and interests as key components of motivation, which subsequently influence achievements and behavioral outcomes (Changlek and Palanukulwong, 2015; Zhao et al., 2018; Hagger and Hamilton, 2019; Shirvan and Alamer, 2022). Based on the Self-Determination Theory (SDT; Deci and Ryan, 1985; Ryan and Deci, 2000), intrinsic motivation involves engaging in activities for inherent satisfaction or interest, whereas extrinsic motivation is driven by external rewards or recognition. On the other hand, amotivation refers to the absence of motivation. The study by Karlen et al. (2019) showed that students with higher levels of both grit dimensions (i.e., perseverance of effort and consistency of interests) tend to be motivated both intrinsically and extrinsically. Several other studies have also identified a strong association between high levels of grit and intrinsic motivation (Chen and Caza, 2018; De La Cruz et al., 2021; Lozano-Jiménez et al., 2021). This body of evidence appears to indicate that students with high levels of grit dimensions, along with intrinsic and extrinsic motivation, are likely to exhibit strong goal-oriented behaviors aimed at achieving long-term objectives, as previously demonstrated by the strong associations found between motivation and goal orientations (Ntoumanis, 2001; Harwood et al., 2015).

The relationship between motivation and achievement goal orientations is often conceptualized through various motivational mechanisms that suggest a causal link (Ntoumanis, 2001; Vansteenkiste et al., 2014). Achievement Goal Theory (AGT; Ames, 1995; Elliot and Church, 1997; Dweck and Leggett, 1998) posits that understanding students’ academic motivation and behaviors requires an examination their underlying goals and purposes during educational activities. Students’ goal orientations, whether mastery-approach (e.g., focusing on understanding and mastering the subject), performance-approach (e.g., aiming to excel beyond peers and gain recognition), or performance-avoidance (e.g., driven by avoiding failure and negative outcomes), significantly influence their motivational drives (Elliot and Church, 1997; Elliot, 1999). Studies utilizing SDT (Deci and Ryan, 1985) and AGT (Elliot and Church, 1997) have explored motivational mechanisms, showing significant interactions between the two theories (Ntoumanis, 2001; Jiang and Zhang, 2021). This suggests that motivation and goal orientations may each precede the other in the motivational process. From a theoretical perspectives, for instance, students’ actions may initially be driven by their enjoyment of the learning process (intrinsic motivation), leading them to focus more on understanding the material (mastery-approach goal; Ciani et al., 2011). Conversely, being primarily focused on learning (mastery-approach goal) can foster activities performed for their inherent satisfaction (intrinsic motivation; Hulleman et al., 2010). This connection is also plausible between extrinsic motivation and performance-oriented goals. Both theories emphasize the role of the social environment in guiding achievement behavior (Ntoumanis, 2001; Jiang and Zhang, 2021). The dynamic interplay between motivation and goal orientations highlights the need for further comparative model analysis to clarify these reciprocal influences.

While several studies have explored the associations between motivation and goal orientations and their impact on learning outcomes and behavioral engagements (Usán Supervía et al., 2019; Chu et al., 2021), to date, comprehensive research exploring the causal relationships within these frameworks, especially concerning the grit dimensions, remains scarce. Only one study investigated the integration of grit dimensions into this motivational mechanism. For example, Karlen et al. (2019) found that an in-depth exploration of grit dimensions, as influenced by implicit theories, predicted goal orientations, which subsequently affected intrinsic and extrinsic motivation and academic achievement among high school students. Their study indicated that perseverance of effort strongly correlates with mastery-approach goals, performance-approach goals, and intrinsic motivation, whereas consistency of interests is closely linked to performance-approach, performance-avoidance goals, and extrinsic motivation. Notably, perseverance of effort, rather than consistency of interests, emerged as the predictor of academic achievement, mediated by adaptive learning goals and intrinsic motivation. However, there is limited information available on the motivational mechanism that include grit dimensions in context of PETE.

The relevance of grit becomes more pronounced when considering the reciprocal relationship between motivation and achievement goals; both factors have been shown to influence key life decisions, such as initiating action or choosing a career path, especially within the teacher education settings (Gorozidis and Papaioannou, 2011, 2014). The substantial impact of grit on motivation and achievement goal orientations underscores its importance as a catalyst in shaping the future intentions of students. In this regard, investigating the career intentions of preservice teachers would be vital to improve retention rates and address the ongoing decline in teacher education student retention (Knox, 2022; Kim, 2023). This issue is also pertinent in the context of PPETs, given the significant concern about retention rates in PETE programs (Keating et al., 2017; Woods and Ayers, 2019). The literature on the associations between grit and career-related outcomes among college students exhibited stronger relationships, indicating that students with higher girt are more likely to remain committed to their chosen major/college and profession (Robertson-Kraft and Duckworth, 2014; Bowman et al., 2015; Butz et al., 2018). Such evidence suggests that grit could play a crucial role in both academic perseverance and professional development.

**Figure 1:**
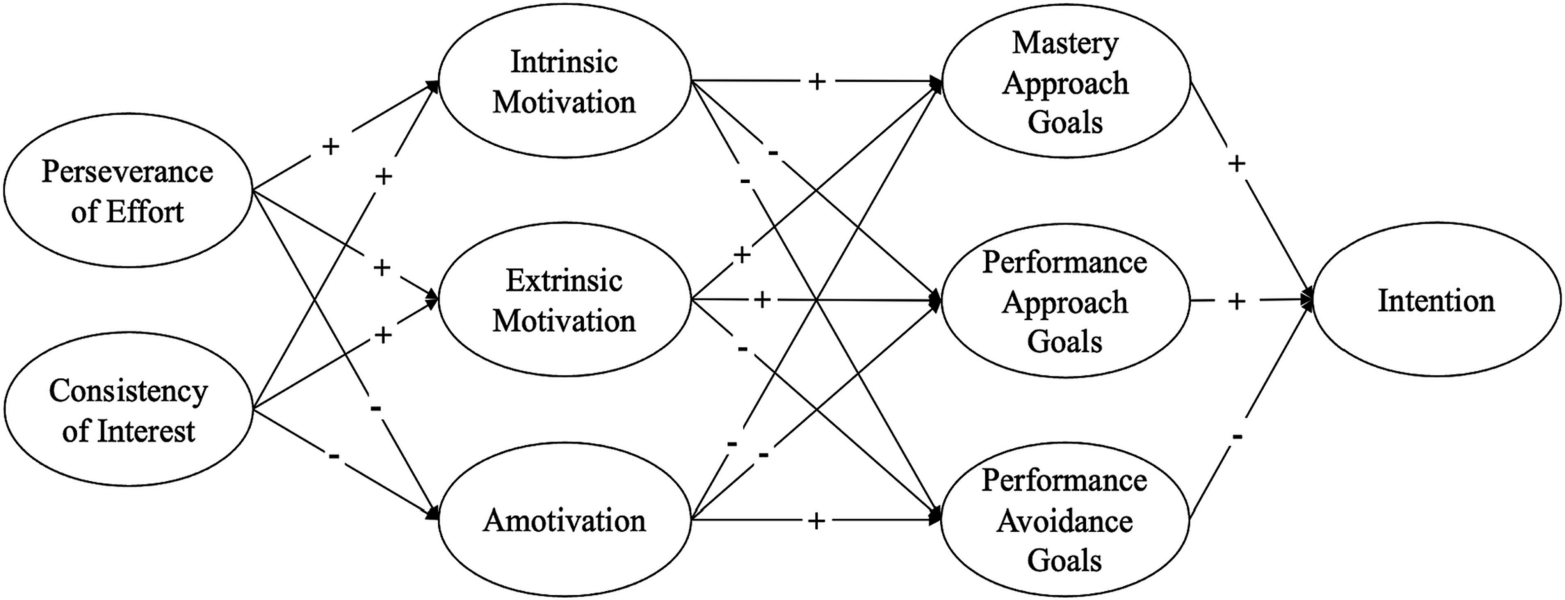
A hypothesized model 1 of the study.

**Figure 2:**
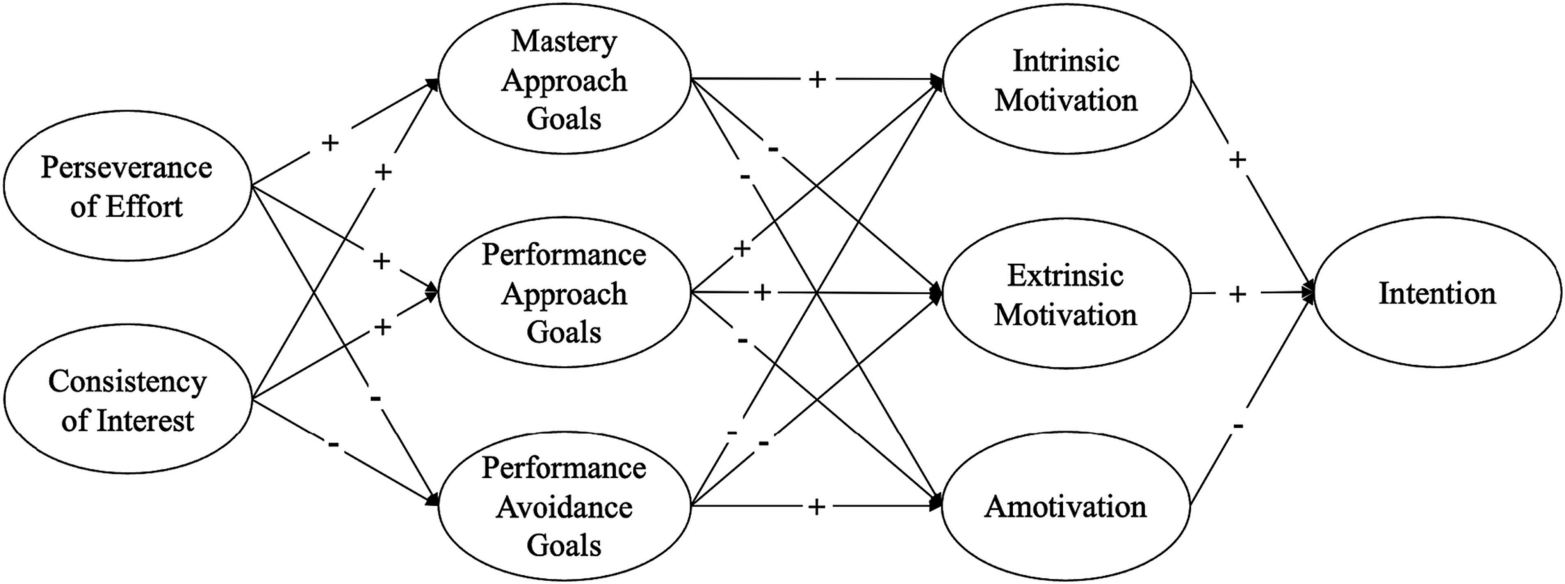
A hypothesized model 2 of the study.

This study introduces motivational mechanisms that explore the interactions among grit, motivation, achievement goals, and intention in PETE students. Figures 1, 2 illustrate the hypothesized models, providing a comprehensive map of this study by delineating both the positive and negative impacts on the variables examined. We explored these two models by varying the sequences of motivation and achievement goals in relation to the grit dimensions and career intention. It was hypothesized that PETE students with higher levels of grit dimensions, either perseverance of effort or consistency of interest, would exhibit higher intrinsic motivation and mastery-approach goals, which in turn would lead to a stronger intention to become PE teachers in the near future. Conversely, students with lower levels of grit dimensions were hypothesized to have higher levels of amotivation or extrinsic motivation, as well as performance-approach and performance-avoidance goals, which could consequently diminish their intention to pursue careers as PE teachers.

Grit, as a trait-level personality construct, has been widely used as a predictor in diverse fields such as academia, workplace, military, and athletic performance (Eskreis-Winkler et al., 2014; Cosgrove et al., 2018; Albert et al., 2021; De La Cruz et al., 2021; Harner et al., 2021). However, given the clear distinct dimensions observed in previous studies, it is recommended to assess grit components individually rather than as a composite measure (Bowman et al., 2015; Hagger and Hamilton, 2019; Karlen et al., 2019; Alhadabi and Karpinski, 2020). By comparing each dimension of grit, we could gain a deeper understanding of why some students exhibit stronger motivations and goals orientations, which in turn contribute to their career intentions. Notably, the specific dimensions of grit (i.e., perseverance of effort and consistency of interests) have been understudied in the context of PETE. Furthermore, existing research highlights the need for further investigation into how grit influences career intentions through specific types of motivational states (i.e., intrinsic motivation, extrinsic motivation, and amotivation) and achievement goal orientations (i.e., mastery and performance approach or avoidance). This necessity is underscored by previous studies’ tendency to focus on limited aspects of the motivational spectrum, often emphasizing intrinsic motivation (Chen and Caza, 2018; Lozano-Jiménez et al., 2021) while neglecting factors like amotivation (Karlen et al., 2019), or concentrating only on one dimension of the motivational mechanism, such as achievement goal orientations (Muenks et al., 2018; Alhadabi and Karpinski, 2020; Liu et al., 2023). Given this, there is a significant need for further investigation to explore the gaps and lack of specificity in our current understanding of how grit dimensions interact with various motivational statuses and achievement goal orientations. Future research is thus warranted to unravel these nuanced relationships and their contextual dependencies.

Despite the growing acknowledgement of the importance of the PETE programs and the need for well-prepared PE teachers, there appears to be limited knowledge regarding the motivational mechanisms underlying PETE major students’ grit and its relationship to motivation, achievement goals, and intention to become PE teachers. Specifically, the interplay between grit dimensions, motivation, achievement goals, and the intention to become a PE teacher remains underexplored. Such a gap is particularly significant given the distinct challenges and opportunities inherent in PETE programs, which can vary in terms of curriculum, training intensity, and expected career outcomes. Understanding these motivational mechanisms is essential for both educators and policymakers aiming to foster grit and other beneficial traits in future teachers. Therefore, the purpose of this study was to investigate the influence of grit dimensions in preservice PE teachers on their motivations and achievement goals, and how these factors might in turn determine their intentions to pursue careers in PE teaching.

The research questions guiding this study were as follows: (i) What is the underlying structural relationship between grit dimensions, motivational factors, achievement goal orientations, and intention to become a teacher among PETE major students? (ii) To what extent do grit dimensions, motivational factors, and achievement goals collectively account for the variance in the intention to pursue a career as a PE teacher among PETE major students?

## 2 Methods

### 2.1 Participants and procedures

In this study, a cross-sectional design was used in conjunction with random sampling. Following ethical approval, recruitment flyers were distributed to faculty members affiliated with PETE programs at universities throughout South Korea. Students enrolled in a 4-year bachelor’s or master’s PETE programs at five regional universities consented to participate in the study. A total of 279 PPETs—comprising 194 males (69.5%) and 85 females (30.5%)—completed online questionnaires. In terms of academic year, the distribution was as follows: 38 freshmen (13.6%), 64 sophomores (22.9%), 57 juniors (20.4%), 45 seniors (16.1%), and 75 graduate students (26.9%). The attained sample size of 279 participants provides sufficient statistical power, exceeding the recommended minimum of 200 samples for the Monte Carlo technique (Stone and Sobel, 1990). This study complied with the Strengthening the Reporting of Observational Studies in Epidemiology (STROBE) guidelines.

### 2.2 Measures

#### 2.2.1 Grit

The Grit Scale-Short Version (Grit-S; Duckworth and Quinn, 2009), comprising eight items, was employed to measure the grit of the students in this study. This scale is structured around two key dimensions: perseverance of effort and consistency of interests. Each dimension in the original scale was represented by four items. However, following an exploratory factor analysis, one item from each dimension was removed due to low factor loading, indicating weak statistical correlation with the corresponding latent variable. Therefore, in our study, both perseverance of effort and consistency of interest were assessed using three items each. Sample items on the Girt-S include “I finish whatever I begin” which assesses perseverance of effort, and “I often set goals but later choose to pursue a different one” addressing consistency of interest. Items related to consistency of interest were reverse-coded. For assessing responses, the Grit-S utilizes a 5-point Likert scale, with response options ranging from 1 (strongly disagree) to 5 (strongly agree). The Grit-S has demonstrated sufficient internal reliability, with Cronbach’s alpha (α) values ranging from 0.74 to 0.79 for the dimensions of consistency of interests and perseverance of effort in educational settings (Alhadabi and Karpinski, 2020; Choi and Seo, 2020). In this study, the reliability (α) for these dimensions was found to be acceptable, ranging between 0.70 and 0.73.

#### 2.2.2 Motivation

This study employed the Academic Motivation Scale (AMS; Vallerand et al., 1992) to examine the diverse motivational factors among PPETs in academic settings. The AMS is structured into three domains: intrinsic motivation (e.g., inherent interest in the subject matter), extrinsic motivation (e.g., external rewards or social approval), and amotivation (e.g., lack of motivation or interest). Sample questions include “I chose this because I wanted to learn about PE” in the intrinsic motivation domain, “I selected the PE major to gain social recognition” in the extrinsic motivation domain, and “I feel like I am wasting my time at school” in the amotivation domain. Each domain consists of three items. The AMS incorporates a 5-point Likert scale, with options ranging from 1 (strongly disagree) to 5 (strongly agree). Previous research has indicated high reliability for the AMS across its three domains, with Cronbach’s alpha (α) values ranging from 0.84 to 0.86 among PPETs (Spittle et al., 2009). In our study, we observed similarly high reliability (α) for the various types of motivation, with values ranging from 0.81 to 0.97.

#### 2.2.3 Achievement goals

Achievement Goal Orientation (AGO) scale (Elliot and Church, 1997) was utilized to evaluate the achievement goal orientations of PPETs in this study. The AGO scale comprises three distinct goal orientations: mastery approach, which focuses on learning and self-improvement (e.g., “My aim is to completely master the material presented in this class”); performance approach, emphasizing outperforming peers (e.g., “I strive to perform well compared to others”); and performance avoidance, which involves an aversion to failure or negative evaluation (e.g., “My goal is to avoid performing worse than other students”). Before beginning the questionnaire, a brief prompt was included to guide students’ focus toward their experiences and objectives specific to their PE courses and major. Respondents rated their agreement with each item using a 5-point Likert scale, where 1 represents “strongly disagree” and 5 indicates “strongly agree.” A previous study demonstrated that the AGO scale has high reliability among university students, with Cronbach’s alpha (α) values ranging from 0.80 to 0.90 (Alhadabi and Karpinski, 2020). The sample in this study demonstrated acceptable reliability coefficients (α), ranging from 0.72 to 0.85.

#### 2.2.4 Intention to become PE teachers

Drawing upon a previous study that examined the intention to remain in the teaching profession among pre-service teachers (Bruinsma and Jansen, 2010), this study assessed the intentions of PPETs to pursue careers as PE teachers in the near future. The assessment focused on three specific questions: “I am committed to pursuing a career as a PE teacher to positively influence young lives through PE,” “I am confident in my ability to adapt and innovate teaching methodologies to engage all students in PE classes,” and “I envision myself as a future PE teacher, actively promoting inclusivity and equal opportunities within the field of PE.” Data were collected using a 5-point Likert scale, ranging from 1 (strongly disagree) to 5 (strongly agree). The scores for the three items were aggregated and their mean was calculated to generate a composite intention variable. High reliability was shown with Cronbach’s alpha (α) of 0.87.

### 2.3 Data analyses

We screened for missing values and found none within our sample set. Initially, we conducted descriptive statistical analyses, assessed variable normality, and calculated correlations using IBM SPSS Statistics 29.0. Correlation strengths (*r*) were classified as follows: weak (≥ 0.1), moderate (≥ 0.4), and strong (≥ 0.7; Dancey and Reidy, 2007). Reliability estimates for the questionnaire items were evaluated through both Cronbach’s alpha (α) and composite reliability (CR), using a threshold of 0.70 (Cronbach, 1951). Subsequently, validity estimates were assessed by calculating the average variance extracted (AVE) values, using a criteria of 0.50 (Fornell and Larcker, 1981). All variables were found to be sufficiently reliable (Table 1). To evaluate the measurement model’s goodness-of-fit, structural equation modeling (SEM) techniques were employed using IBM AMOS 26.0. Model fit was assessed based on the following indices and corresponding cut-off values: normed chi-square (*χ*^2^/df), root mean square error of approximation (RMSEA; < 0.08), incremental fit index (IFI; > 0.90), Tucker-Lewis index (TLI; > 0.90), and comparative fit index (CFI; > 0.90; Hu and Bentler, 1999). In addition, to test for mediating effects, a bootstrapping procedure was conducted with 5,000 bootstrap samples, and the results were interpreted within a 95% confidence interval (CI). Participants’ sex and academic year were controlled as extraneous variables in the model by linking them as covariates to both grit dimensions and to the endogenous variables (e.g., motivational factors, goal orientations, and career intentions). For all analyses, the significance level (value of *p*) was set at 0.05.

**Table 1:** Summary of Cronbach’s alpha, CR, AVE values.

Factors/Items	λ	α	CR	AVE
Perseverance of effort		0.73	0.91	0.76
I finish whatever I begin	0.78			
Setbacks do not discourage me	0.77			
I am diligent	0.75			
Consistency of interest		0.70	0.89	0.74
I often set goals but later choose to pursue a different one	0.77			
I have been obsessed with a certain idea or project for a short time but later lost interest	0.76			
New ideas and projects sometimes distract me from previous ones	0.73			
Intrinsic motivation		0.81	0.95	0.85
I chose PE major because I am interested in this field	0.87			
I chose PE major because I wanted to engage in specialized studies related to PE	0.80			
I chose PE major because I think the work in this field will be enjoyable	0.77			
Extrinsic motivation		0.97	0.88	0.72
I chose PE major because it is related to the career I aspire to have	0.96			
I chose PE major because I think it will earn me social recognition	0.96			
I chose PE major because it has a positive social image	0.81			
Amotivation		0.85	0.96	0.89
I feel like I am wasting my time at school	0.86			
I do not know what I am learning in class	0.82			
Honestly, I do not know why I am attending class.	0.81			
Mastery approach goals		0.81	0.84	0.64
My aim to completely master the material presented in this class	0.76			
I am striving to understand the content of this course as thoroughly as possible	0.75			
My goal is to learn as much as possible	0.74			
Performance approach goals		0.85	0.85	0.66
My goal is to get better grades compared to other students	0.85			
I am making an effort to get better grades than other students	0.84			
My goal is to perform better than the other students	0.79			
Performance avoidance goals		0.72	0.80	0.63
My goal is to avoid performing worse than other students	0.84			
I am striving to not get lower grades than others	0.81			
My goal is to avoid getting lower grades compared to other students	0.76			
Intention		0.87	0.93	0.77
I am committed to pursuing a career as a PE teacher to positively influence young lives through PE.	0.92			
I am confident in my ability to adapt and innovate teaching methodologies to engage all students in PE classes.	0.86			
I envision myself as a future PE teacher, actively promoting inclusivity and equal opportunities within the field of PE.	0.84			

Items related to the consistency of interest in the Grit scale were reverse-coded.

## 3 Results

Table 2 presents the Pearson product–moment correlations, along with the means and standard deviations, for each scale related to the variables under investigation in this study. The mean scores for all variables under study deviated from the midpoint of the 5-point Likert scale.

**Table 2:** Correlations, means, and SD among grit, motivation, achievement goal, and intention.

Scales		1	2	3	4	5	6	7	8	9
1.	Perseverance of effort									
2.	Consistency of interest	0.343**								
3.	Intrinsic motivation	0.224**	0.187**							
4.	Extrinsic motivation	0.017	0.053	0.212**						
5.	Amotivation	−0.283**	−0.266**	−0.358**	−0.119*					
6.	Mastery approach goal	0.382**	0.380**	0.302**	0.276**	−0.425**				
7.	Performance approach goal	0.254**	0.207**	0.060	0.251**	−0.163*	0.416**			
8.	Performance avoidance goal	0.014	0.056	−0.017	0.139*	0.035	0.092	0.360**		
9.	Intention	0.104	0.184**	0.249**	0.169**	−0.248**	0.382**	0.183**	**−0.058**	
	*M*	4.49	4.50	4.67	4.01	4.65	3.99	3.73	3.14	4.16
	*SD*	0.43	0.43	0.43	1.13	0.43	0.79	0.88	0.92	0.63

Values in bold indicate statistical significance: *p < 0.05, **p < 0.01.

The analysis of the relationships between various psychological constructs revealed a range of correlation strengths among the scales, ranging from weak to moderate (0.119–0.425), and including both positive and negative correlations. Specifically, the results revealed a statistically significant positive correlation between perseverance of effort and consistency of interests (*r* = 0.343, *p* < 0.01). Grit variables (perseverance of effort and consistency of interests) were positively associated with intrinsic motivation (*r* ranging from 0.187 to 0.224, *p* < 0.01), mastery approach goals (*r* ranging from 0.380 to 0.382, *p* < 0.01), and performance approach goals (*r* ranging from 0.207 to 0.254, *p* < 0.01). These variables were negatively correlated with amotivation (*r* ranging from −0.266 to −0.283, *p* < 0.01). Notably, only consistency of interests was statistically and positively correlated with career intensions (*r* = 0.184, *p* < 0.01).

Intrinsic motivation exhibited a positive correlation with extrinsic motivation (*r* = 0.212, *p* < 0.01), while amotivation was negatively correlated with both intrinsic motivation and extrinsic motivation (*r* ranging from −0.119 to −0.358, *p* < 0.01). Mastery approach goal was positively correlated with both intrinsic and extrinsic motivation (*r* ranging from 0.276 to 0.302, *p* < 0.01), but negatively associated with amotivation (*r* = −0.425, *p* < 0.01). Performance approach goal was positively associated with mastery approach goal (*r* = 0.416, *p* < 0.01) and extrinsic motivation (*r* = 0.251, *p* < 0.01), yet negatively related to amotivation (*r* = −0.163, *p* < 0.01). Performance avoidance goal displayed a positive correlation with extrinsic motivation (*r* = 0.139, *p* < 0.05). and performance approach goal (*r* = 0.360, *p* < 0.01).

The intention to become a PE teacher in the future was positively related to intrinsic motivation, extrinsic motivation, mastery approach goal, and performance approach goal (*r* ranging from 0.169 to 0.382, *p* < 0.01), while it was negatively associated with amotivation (*r* = −0.248, *p* < 0.01). Some variable pairs yielded non-significant correlations (*r* ranging from *0*.014 to 0.104, *p* > 0.05).

**Figure 3:**
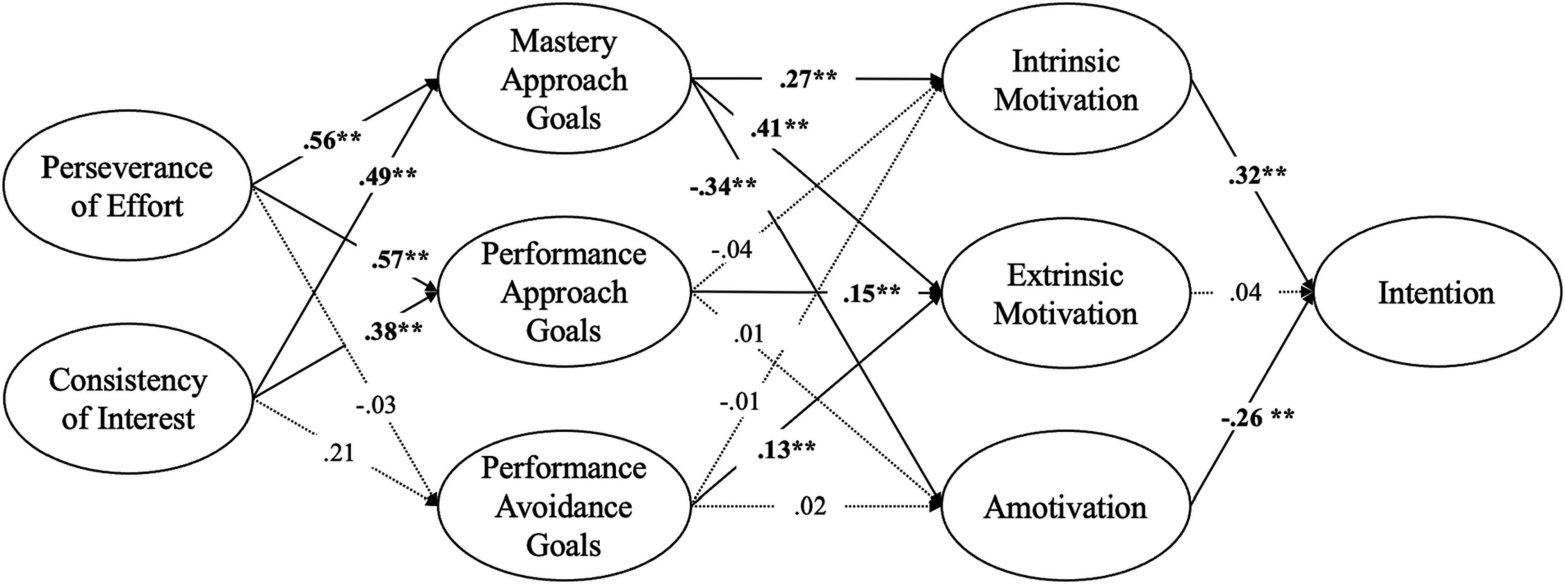
Results of path analysis for the final model in the study. **p < 0.01.

Our analysis utilizing SEM confirmed that the hypothesized model 1 met the goodness-of-fit criteria, with all relevant indices satisfying their respective benchmarks (χ^2^/df = 2.053, RMSEA = 0.062, IFI = 0.92, TLI = 0.91, CFI = 0.92). Similarly, the hypothesized model 2 demonstrated an adequate goodness-of-fit (χ^2^/df = 1.928, RMSEA = 0.058, IFI = 0.92, TLI = 0.91, CFI = 0.92). However, a closer examination through direct and indirect path analysis revealed distinct differences between the models. In the model 1, a significant impact was observed solely from the consistency of interests. In contrast, the model 2 showed that both perseverance of effort (*β* = 0.10, 95% CI [0.04, 0.28], *p* < 0.01) and consistency of interests (*β* = 0.10, 95% CI [0.10, 0.19], *p* < 0.05) influenced career intention via a motivational mechanism. Given these insights and the central role of grit dimensions as predictors, we chose the model 2 as our final model. Figures 2, 3 illustrates the final model based on the results of our path analysis. During model testing, we found that the extraneous variables (sex and academic year) did not have statistically significant associations with the study variables (*p* > 0.05), indicating that sex and academic year did not confound the relationships.

Both perseverance of effort and consistency of interest positively affected mastery approach goals (*β* ranging from *0*.49 to 0.56, *p* < 0.01), performance approach goals (*β* ranging from 0.38 to 0.57, *p* < 0.01), but were not a statistically significant predictor on performance avoidance goal (*p* > 0.05). The achievement goal orientations factors showed significant predictors on the motivation. For instance, mastery approach goals were strong predictors across all motivational variables (*β* ranging from −34. to 0.41, *p* < 0.01). Both performance approach and avoidance goals were found to similarly influence extrinsic motivation (performance approach goals: *β* = 0.15, *p* < 0.01; performance avoidance goals: *β* = 0.13, *p* < 0.01), yet neither type of performance goal significantly predicted intrinsic motivation or amotivation (*p* > 0.05). In examining the relationship between motivational factors and intention, it was found that intrinsic motivation significantly influenced intention (*β* = 0.32, *p* < 0.01), while amotivation had a negative influence on intention (*β* = −0.26, *p* < 0.01). In contrast, extrinsic motivation did not significantly affect intention (*p* > 0.05).

The analysis of indirect effects uncovered complex structural relationships among grit dimensions, achievement goals, motivational factors, and the intention to pursue a career in PE teaching. As previously mentioned, both perseverance of effort and consistency of interests significantly influenced intention indirectly (*p* < 0.05–0.01). Specifically, these two dimensions of grit showed statistically significant indirect effects on intrinsic motivation (perseverance of effort: *β* = 0.13, 95% CI [0.05, 0.34], *p* < 0.01; consistency of interests: *β* = 0.12, 95% CI [0.04, 0.24], *p* < 0.01) and amotivation (perseverance of effort: *β* = −0.19, 95% CI [−0.41, −0.07], *p* < 0.01; consistency of interests: *β* = −0.16, 95% CI [−0.32, −0.04], *p* < 0.01). However, only consistency of interests demonstrated indirect effects on extrinsic motivation (*β* = 0.29, 95% CI [0.01, 0.90], *p* < 0.01). Interestingly, while mastery approach goals showed statistically significant indirect effects on intention (*β* = 0.20, 95% CI [0.11, 0.31], *p* < 0.01), performance approach or avoidance goals did not (*p* > 0.05). These results indicate that both perseverance of effort and consistency of interests are intrinsically linked to mastery approach goals, which play a crucial role in fostering intrinsic motivation and shaping the intention to become a PE teacher.

## 4 Discussion

The study aimed to examine the motivational mechanisms that underlie PETE students’ level of grit dimensions and to investigate how these mechanisms are associated with their motivation, achievement goal orientations, and intentions to pursue a career in PE teaching. The findings support the final model, revealing a range of weak to moderate correlations among the variables—both positive and negative—and confirming the goodness-of-fit in the model.

The results of this study indicated that PETE students with higher levels of grit dimensions, including perseverance of effort and consistency of interests, tend to have a positive association with intrinsic motivation and mastery/performance approach goals, while being negatively related to amotivation. These findings reinforce the significant role of both perseverance of effort and consistency of interests in fostering beneficial motivational and goal-oriented behaviors in education, consistent with previous research (Muenks et al., 2018; Karlen et al., 2019; Alhadabi and Karpinski, 2020; Lozano-Jiménez et al., 2021). However, the impact of each grit dimension on motivation and goal orientations has shown complex and varied outcomes in prior studies. For example, while Karlen et al. (2019) found that both dimensions were related to extrinsic motivation, our study did not observe this relationship. Although our findings indicated a significant link between both grit dimensions and mastery/performance approach goals, other research (Muenks et al., 2018; Alhadabi and Karpinski, 2020) demonstrated that perseverance of effort, rather than consistency of interests, was more strongly associated with these goal orientations. The contradictory findings could be due to differences in the study samples, which covered a wide educational spectrum, from high school to university students, and included general education majors, rather than focusing exclusively on PETE. The unique nature of PETE programs, which often engage students more directly in physical activities and sports demanding both perseverance of effort and consistency of interest, could be a key factor contributing to these differing results. The practical and physically demanding nature of the PETE curriculum may foster or require a different set of motivational characteristics and goal orientations (Poteliūnienė et al., 2022).

Our study revealed that only consistency of interests had a statistically significant positive correlation with intention to become a PE teacher, suggesting a nuanced role for different facets of grit in shaping career intentions. This result was in line with the previous evidence (Bowman et al., 2015), which found that consistency of interests alone was statistically and negatively related to intention to change careers. This suggests that students who maintain a consistent interest in their field of study or career path are less likely to consider changing careers. This outcome could be particularly relevant in the context of PETE, where commitment to a physically and educationally demanding discipline is crucial for long-term career satisfaction and persistence. Studies indicated a prominent role for consistency of interests in environments that demand high engagement in physical activities and sports participation (Hein et al., 2019; Newland et al., 2020). Consistency of interests might therefore reflect a deeper alignment with the core values and demands of the PE field, reinforcing the likelihood of a sustained commitment to this career path. However, it is noteworthy that the path analysis revealed a broader dynamic, also highlighting perseverance of effort as an equally important predictor, playing a significant role in career intention when viewed through the lens of motivational mechanisms.

The analysis of direct and indirect effects deepens our understanding of the intricate relationships among the studied variables, underscoring the pivotal roles of grit dimensions in influencing career intentions via motivational mechanisms. Students with high levels of both perseverance of effort and consistency of interests are likely to be closely aligned with mastery approach goals, a crucial factor in nurturing intrinsic motivation and shaping their aspiration to become PE teachers. Consistent with our findings, Karlen et al. (2019)‘s study also demonstrated that perseverance of effort significantly impacts mastery approach goals and intrinsic motivation, which subsequently influences academic achievement. However, unlike their study, our findings additionally revealed a notable role for consistency of interest in these relationships. This discrepancy may be explained by differences in age groups, educational contexts, and cultural backgrounds, as the previous study focused on Swiss secondary school students (Karlen et al., 2019). Notably, prior research in movement and sport-related contexts has identified consistency of interest as a stronger predictor (Hein et al., 2019; Newland et al., 2020). In PETE majors, where the integration of academic study and PE is essential, the influence of grit dimensions on career intentions might be channeled through varied motivational pathways. For example, the physical challenges and team-oriented activities inherent in PETE programs may foster a stronger link between perseverance of effort and consistent interest in PE. In addition, the hands-on experiences and fieldwork in PETE can shape how students internalize their goal orientations and motivation, crucial for their commitment to a future profession in this field. Consequently, this unique context may result in distinct patterns of how grit dimensions affect career intentions through achievement goals and motivation, differing from those observed in more traditional academic environments.

The enhanced mediating role of mastery-approach goals in shaping career intentions suggests that PETE programs may benefit from interventions aimed at enhancing mastery-approach goals, such as providing more opportunities for skill development, emphasizing learning and personal improvement over competition, and fostering a classroom environment that values mastery and personal progress. Research informed by the AGT (Ames, 1995; Elliot and Church, 1997; Dweck and Leggett, 1998) also supports the notion that mastery approach goals, with their intrinsic focus on learning and self-improvement, are linked to sustainable and effective long-term engagement and achievement (Harackiewicz et al., 2000; Pekrun et al., 2006; Benita and Matos, 2021). Given that the current study among PPETs demonstrated that both dimensions of grit positively influenced mastery approach goals—which in turn shape intrinsic motivation—PETE programs could potentially benefit from incorporating grit into their pedagogical strategies. Specifically, faculty and administrators might focus on methods that develop mastery approach goals to enhance PPETs’ long-term career commitment. Furthermore, future research is warranted to explore interventions or educational strategies that effectively cultivate and sustain such interests, which could lead to more stable career trajectories in the field of PE.

### 4.1 Limitation

This study presents several limitations that warrant consideration. Firstly, the cross-sectional design inherently constrained the capacity to establish causal relationships among the study variables. Future research should consider longitudinal designs to capture the temporal dynamics of these relationships and to allow for causal inferences. Secondly, the study’s sample was restricted to PPETs in South Korea. Given the observed discrepancies in findings across various studies, which may be attributed to differences in age groups, academic majors, and cultural contexts, future research should endeavor to replicate these results in more diverse populations. Lastly, while we controlled for sex and academic year as the extraneous variables, it is crucial to acknowledge that other confounding factors, such as economic conditions or job market trends for PE teachers in South Korea, might also influence the relationships among grit, goal orientations, motivation, and career intentions.

## 5 Conclusion and practical implications

This study contributes to the literature by exploring the interplay between grit, achievement goals, motivation, and career intentions in the context of PETE programs, which has not been explored. Our study uncovered significant interrelations among grit dimensions, motivational mechanism, and career intentions among PPETs. The finding underscores the importance of nurturing and supporting both grit dimensions in PETE program. We discovered that both perseverance of effort and consistency of interests positively correlated with intrinsic motivation and mastery/performance approach goals, while exhibiting an inverse relationship with amotivation. Consistency of interests alone was notably associated with the intention to pursue a career in PE teaching. Furthermore, mastery-approach goals were identified as significant influencers of motivational factors. The intention to become a PE teacher was significantly and positively linked with mastery approach goals, in contrast to a negative association with amotivation. The SEM analysis validated our hypothesized model, shedding light on the intricate effects of grit dimensions on motivational constructs. Indirect effects analysis highlighted the pivotal role of both grit dimensions and mastery approach goals in shaping career intentions among future PE teachers. This insight contributed to empirical evidence supporting the integral influence of grit dimensions and motivational mechanism in shaping the career intentions of future PE teachers. Echoing prior research (Duckworth et al., 2009; Robertson-Kraft and Duckworth, 2014; Keesey et al., 2018), our study reinforced the notion that grit is a pivotal component in the realm of teacher education.

The present study has noteworthy practical implications for educators and policymakers within the realm of PETE. Specifically, interventions designed to foster grit dimensions and mastery-approach goals could lead to beneficial outcomes, such as the enhancement of intrinsic motivation and the strengthening of career intentions among PETE students. For instance, the curriculum could incorporate activities that build grit—challenging students to develop both perseverance of effort and consistency of interest—alongside applications of mastery-approach goals in classroom settings (Phan, 2010; Emery et al., 2018). In addition, the establishment of mentorship programs, where senior students or professionals in the field guide junior students, and the inclusion of project-based learning activities, which require a long-term commitment and thus the cultivation of grit, could provide practical avenues for skill and trait development (Ambrosetti and Dekkers, 2010). Furthermore, training programs for faculty and administrators in the PETE program are warranted (Duckworth et al., 2009; Robertson-Kraft and Duckworth, 2014; Keesey et al., 2018). These could take the form of workshops and seminars that offer both theoretical and practical frameworks for recognizing and cultivating grit. Comprehensive efforts are likely to foster grit in PETE students and simultaneously prepare them to surmount the inevitable challenges of their professional trajectories.

## Data availability statement

The raw data supporting the conclusions of this article will be made available by the corresponding author, without undue reservation.

## Ethics statement

The studies involving humans were approved by the Institutional Review Board (IRB) of Pusan National University. The studies were conducted in accordance with the local legislation and institutional requirements. The participants provided their written informed consent to participate in this study.

## Author contributions

JL: Conceptualization, Methodology, Supervision, Validation, Writing – original draft, Writing – review & editing. JP: Conceptualization, Formal analysis, Methodology, Project administration, Resources, Validation, Writing – original draft, Writing – review & editing.
